# Osteosarcopenia as a risk factor for depression: Longitudinal findings from the SHARE study

**DOI:** 10.1016/j.bonr.2025.101848

**Published:** 2025-05-05

**Authors:** Nicola Veronese, Francesco Saverio Ragusa, Shaun Sabico, Ligia Juliana Dominguez, Mario Barbagallo, Gustavo Duque, Lee Smith, Nasser Al-Daghri

**Affiliations:** a Geriatric Unit, Department of Internal Medicine and Geriatrics, University of Palermo, 90127 Palermo, Italy; b Chair for Biomarkers of Chronic Diseases, Biochemistry Department, College of Science, King Saud University, Riyadh 11451, Saudi Arabia; c Department of Medicine and Surgery, Kore University of Enna, 94100 Enna, Italy; d Bone, Muscle & Geroscience Group, Research Institute of the McGill University Health Centre, Montreal, QC, Canada; e Dr Joseph Kaufmann Chair in Geriatric Medicine, Department of Medicine, McGill University, Montreal, QC, Canada; f Centre for Health Performance and Wellbeing, Anglia Ruskin University, Cambridge, UK

**Keywords:** Osteosarcopenia, Osteoporosis, Sarcopenia, Depression, SHARE

## Abstract

**Background:**

Osteosarcopenia (i.e., the co-existence of osteoporosis and sarcopenia) and depression are highly prevalent among older people. However, the association between osteosarcopenia and depression in older people is largely unknown. Therefore, the present study aims to investigate this possible association in a representative sample of the older adult population in Europe and Israel.

**Methods:**

Osteosarcopenia was defined as the concomitant presence of osteoporosis and sarcopenia; depressive symptoms in the SHARE study were self-reported using the EURO-D scale. The association between the presence of osteosarcopenia at baseline in people free from depression and incident depression during 12 years of follow-up was analyzed using a Cox's regression analysis, adjusting for several baseline covariates.

**Results:**

16,452 participants were included (mean age 63.7, SD 9.6; females 50.6 %). During the follow-up period, 5056 participants (31.1 % of the initial population) became depressed. People affected by osteosarcopenia became depressed in more than half of the cases compared to a quarter of controls. After adjusting for several potential baseline confounding variables, only sarcopenia (HR, hazard ratio = 1.17; 95 % CI, confidence intervals 1.04–1.32; *p* = 0.009) and osteosarcopenia (HR = 1.27; CI 95 % 1.12–1.58; *p* = 0.003) were significantly associated with a higher risk of depression.

**Limitations:**

Definition of sarcopenia using an anthropometric equation; definition of depression using the EURO-D scale.

**Conclusions:**

The present study identified a significant association between osteosarcopenia and depression over 12 years of follow-up, mainly driven by sarcopenia. If future research confirms the present findings, it may then be prudent to target those with osteosarcopenia to aid in the prevention of onset depression.

## Highlights

• If osteosarcopenia is a risk factor for depression is poorly known.
• In our study, the presence of osteosarcopenia increased the risk of depression.
• The combination of osteoporosis and sarcopenia could be considered as a novel risk factor for depression.

## 1 Introduction

The aging population has resulted in a rise in chronic diseases such as diabetes, hypertension, and obesity, as well as various mental health complications such as depression, which pose serious public health problems (Sun and Li, 2023). Depression is among the most common mood disorders in older adults, and it is associated with poor quality of life and increased morbidity, disability and mortality (Rodda et al., 2011); it often remains undetected and untreated (Devita et al., 2022). Negative consequences of late-life depression include increased use of mental health services and mortality rates due to cardiovascular causes, increased cancer rates, and substantially greater risk for suicide (Dines et al., 2014). It is thus of utmost importance to identify older adults who are at greater risk of developing depression or depressive symptoms.

A new geriatric concept, osteosarcopenia, is defined as the concurrent presence of osteoporosis and sarcopenia, and there is increasing interest in this concept among researchers in geriatrics (Kirk et al., 2019). Sarcopenia is defined as loss of muscle mass, strength and function and is common in older adults. Importantly, sarcopenia has been found to be associated with falls, fractures, cardiometabolic diseases, and lower quality of life (Smith et al., 2022). Osteoporosis is characterized by low bone mineral density caused by altered bone microstructure, ultimately predisposing patients to low-impact, fragility fractures. These fractures lead to a significant decrease in quality of life, increasing morbidity, mortality, and disability. The combination of these two entities, i.e., osteosarcopenia is associated with a higher risk of incident falls and fractures, worse life satisfaction and disability in older people (Teng et al., 2021). Assessing and identifying osteosarcopenia is essential for preventing falls and fractures (Lee et al., 2024). The loss of independence, decreased mobility, and increased vulnerability to falls and fractures can contribute to feelings of helplessness, social isolation, and reduced quality of life, all of which are risk factors for depression (Maier et al., 2021).

An Australian study of 680 older people found osteosarcopenia to be associated with a higher presence of depression and malnutrition (Huo et al., 2015). However, participants in this study were older individuals who already had a history of falling, and the study was carried out on a relatively small population sample size. Another study carried out in Korea including 885 participants over aged 60 years, demonstrated how osteosarcopenia exacerbates frailty, disability and depression, thereby increasing susceptibility to various chronic diseases (Park et al., 2021). However, this study was carried out on a small sample of community-dwelling older adults and thus is not likely to be representative. To the best of the authors' knowledge, no other studies have been published on the potential association between osteosarcopenia and depression in larger populations.

Given the progressive nature of osteosarcopenia and its consequences-such as disability, reduced mobility, falls, and social isolation-it is plausible that osteosarcopenia serves as a precursor to depression. The identification of osteosarcopenia as a risk factor for depression has important clinical implications, as early interventions on the musculoskeletal system may help prevent depression later in life. Therefore, this study aims to investigate the longitudinal association between osteosarcopenia and incident depression in a large representative cohort of older adults living in Europe and Israel.

## 2 Materials and methods

### 2.1 Study design

The present analysis used data from multiple waves of the SHARE study, a multidisciplinary and cross-national panel database of micro data on health, socio-economic status, and social and family networks. SHARE is a longitudinal study that includes a representative sample of the European and Israeli populations (http://www.share-project.org/organisation/share-eric.html) (Börsch-Supan et al., 2013).

Briefly, the SHARE study is a cross-national panel study that collects data on health, socioeconomic status, and social networks of individuals aged 50 years and older across European countries and Israel (Börsch-Supan et al., 2013). The study aims to provide insights into the aging process and its implications for policy-making. Participants in the SHARE study must meet the following inclusion criteria (Sun and Li, 2023): Individuals must be aged 50 years or older at the time of recruitment (Rodda et al., 2011); Participants must be members of a private household (Devita et al., 2022); Participants must be able to answer the survey questions themselves; however, in cases of severe cognitive impairment, a proxy respondent (e.g., a close relative) may provide information on their behalf (Börsch-Supan et al., 2013).

The SHARE study primarily targets the general, non-institutionalized population, including healthy individuals as well as those with chronic conditions or disabilities (Börsch-Supan et al., 2013). The inclusion of individuals with varying health conditions allows the study to capture a comprehensive picture of aging across different socioeconomic and health backgrounds. The SHARE study employs a probability-based sampling approach to ensure representativeness across the target population.

In this longitudinal cohort study, we used the data from wave 1 (baseline, between 2004 and 2006), wave 2 (2006–2007), wave 4 (2011/2012), wave 5 (2013), wave 6 (2015), and wave 7 (2017/2018) (Scherpenzeel et al., 2020). The final dataset included the following countries: Austria, Germany, Sweden, the Netherlands, Spain, Italy, France, Denmark, Greece, Switzerland, Belgium, Israel, Czech Republic, Poland, Luxembourg, Hungary, Portugal, Slovenia, Estonia, Croatia, Lithuania, Bulgaria, Cyprus, Finland, Latvia, Malta, Romania, and Slovakia. SHARE follows a longitudinal panel design, where the same individuals are surveyed across multiple waves. To maintain sample representativeness over time, refreshment samples are drawn periodically to replace participants who drop out due to nonresponse or mortality.

Using multistage clustered sampling, the nationally representative SHARE study explores this cross-country setting as a ‘natural laboratory’ across scientific disciplines and, over time, to turn the challenges of population aging into opportunities and provide policymakers with reliable information for evidence-based policies. SHARE data collection is based on computer-assisted personal interviewing (CAPI). The interviewers conducted face-to-face interviews using a laptop on which the CAPI instrument is installed in the native language of the participants.

### 2.2 Standard protocol approvals, registrations, and patient consents

The SHARE study is subject to continuous ethics review. During Waves 1 to 4, SHARE was reviewed and approved by the Ethics Committee of the University of Mannheim. Wave 4 and the continuation of the project were reviewed and approved by the Ethics Council of the Max Planck Society. In addition, the country implementations of SHARE were reviewed and approved by the respective ethics committees or institutional review boards whenever this was required. The numerous reviews covered all aspects of the SHARE study, including sub-projects and confirmed the project to be compliant with the relevant legal norms and that the project and its procedures agree with international ethical standards (http://www.share-project.org/fileadmin/pdf_documentation/SHARE_ethics_approvals.pdf). Written informed consent was obtained from all participants in the study before data were collected.

### 2.3 Exposure: osteosarcopenia

In the SHARE study, body composition was not evaluated with gold standard measures, such as DXA (Dual-Energy X-ray Absorptiometry). Therefore, we used a surrogate measure of low fat-free mass to estimate body composition, which is defined as having a low SMI (skeletal mass index). SMM (skeletal muscle mass) was calculated based on the equation proposed by Lee and colleagues (Lee et al., 2000), i.e.: ASM = 0.244*weight + 7.8*height + 6.6*sex–0.098*age + race–3.3 (where female = 0 and male = 1; race = 0 (White and Hispanic), race = 1.9 (Black), and race = −1.6 (Asian)). Next, SMM was divided by body mass index (BMI) based on weight and height measured by a trained nurse to create the SMI (Studenski et al., 2014). Low SMM was defined as the lowest quartile of the SMI based on sex-stratified values (Tyrovolas et al., 2016). The equation proposed by Lee has been previously used in the European population (Veronese et al., 2023; Veronese et al., 2022; Veronese et al., 2021) and validated against gold standard methods, such as DXA (Carnevale et al., 2018; Villani et al., 2014).

The identification of sarcopenia was completed using low handgrip strength defined as <27 kg for men and <16 kg for women using the maximum value of three handgrip measurements of the dominant hand, as indicator of low muscle strength (Cruz-Jentoft et al., 2019). Grip strength in kilograms was measured using a Smedley dynamometer (TTM; Tokyo, Japan), with the upper arm being held against the trunk and the elbow in a 90-degree flexion (Steptoe et al., 2013).

Osteosarcopenia was defined as the concomitant presence of osteoporosis and sarcopenia. The presence of osteoporosis was ascertained using self-reported information that also included questions about previous hip fracture, as well as medications (hormones or other) for osteoporosis. Then, we divided the participants into osteosarcopenia (both sarcopenia and osteoporosis present), only osteoporosis, only sarcopenia, and controls.

### 2.4 Outcome: depression

Depressive symptoms in the SHARE study were self-reported using the EURO-D scale that was developed by a European consortium at the baseline and during the follow-up assessments (Prince et al., 1999a). This scale contains 12 different items, i.e., depression, pessimism, suicidality, guilt, sleep quality, interest, irritability, appetite, fatigue, concentration (on reading or entertainment), enjoyment, and tearfulness. Full questions and response options are fully reported elsewhere (Maskileyson et al., 2021). The EURO-D scale produces a score ranging from 0 to 12, the number of depressive symptoms determines the score. Consequently, a higher score implies a higher severity of depressive symptoms. A score of EURO-D ≥ 4 is indicative of depression, as proposed by other studies (Portellano-Ortiz et al., 2018; Prajwal et al., 2021; Miller et al., 2021).

### 2.5 Covariates

For assessing the association between osteosarcopenia and the risk of depression, we considered several possible confounding factors at baseline evaluation based on the literature (Polito et al., 2022), including age (as continuous variable), sex, marital status (married and living together with spouse vs. others), educational level (no educational level vs. other degrees), physical activity level (sports or activities that are vigorous, hardly or never vs. other options), alcohol consumption, smoking at present vs. previous/never, country (categorized as mentioned before and used as dummy variable), presence of any chronic medical condition, defined using all the 14 conditions assessed in the SHARE study.

### 2.6 Statistical analysis

Means and standard deviations (SD) were used to describe continuous variables, while percentages were used for categorical variables. Data were reported as means and standard deviation values (SD) for quantitative measures and as percentages for discrete variables. Levene's test was used to test the homoscedasticity of variances, and if its assumption was violated, Welch's ANOVA was used. *P*-values were calculated using the Jonckheere–Terpstra test for continuous variables and the Mantel–Haenszel Chi-square test for categorical variables by the groups of osteosarcopenia.

The association between the presence of osteosarcopenia at wave 1 in people free from depression at baseline and incident depression was analyzed using a Cox's regression analysis, adjusting for previously mentioned confounding variables. The date of the presence of depression was ascertained using the wave in which EURO-D scale >4 was detected or, for those without depression, the date of the last observation. The deceased were right-censored. The factors included in the multivariate analysis were initially based on previous literature (Polito et al., 2022). The collinearity among factors was assessed with the aim of excluding the covariates having a variance inflation factor over two, but no factors considered were excluded for this reason. The results were reported as hazard ratios (HRs) with their 95 % confidence intervals (CIs). Similarly, we did run a linear regression analysis using as dependent variable the values of EURO-D score at the longest evaluation possible and as covariates those indicated in the multivariate Cox's regression analysis and as dependent variables SMM, SMI and handgrip strength. The results were reported as standardized betas with their 95 % CIs.

To test the robustness of our results, we did run several sensitivity analyses, using the factors cited before as strata (in case of age, we used the median value). Moreover, we added a sensitivity analysis about estimated SMM (per SD) as continuous variable with onset of depression in Cox model with adjustment for same covariates in full model, to better understand if the results found for sarcopenia and osteosarcopenia depends on SMM or on obesity, represented by the denominator of the SMI, i.e., BMI.

All statistical analyses were two-tailed, and a *p*-value <0.05 was considered statistically significant using Bonferroni's correction. All analyses were performed using SPSS 26.0 version software.

## 3 Results

**Fig. 1:**
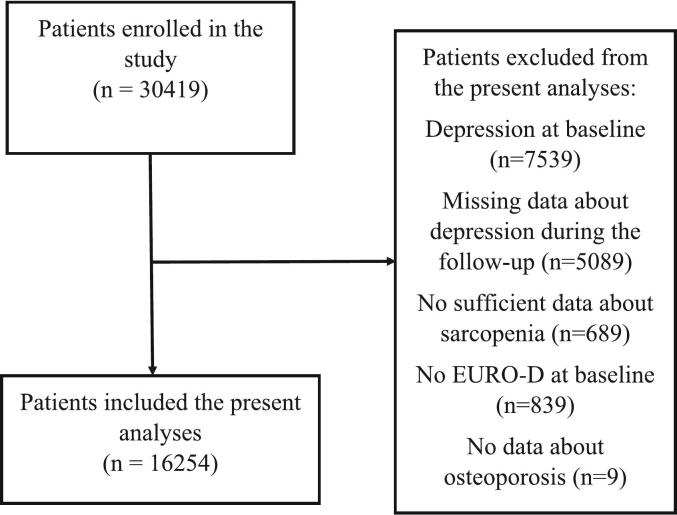
Flow-chart of the study.

Among 30,419 participants initially included in wave 1, as also shown in Fig. 1**,** we excluded 7539 because they had depression at the baseline, 5089 due to missing data about depression during follow-up and 689 since no sufficient data about sarcopenia were available, 893 owing to lack of data on depressive symptoms (no EURO-D done at the baseline), and 9 as there were no data respectively regarding osteoporosis, ultimately leaving 16,254 participants at the baseline evaluation. The 5089 participants lost during follow-up were older and more frequently females than the 16,254 included (*p* < 0.0001 for both parameters), furthermore those lose to follow-up reported higher EURO-D levels at baseline (p < 0.0001).

The 16,254 included participants had a mean age of 63.7 (SD = 9.6) years at baseline, and they were predominantly females (50.6 %).

At baseline, the 16,254 participants were divided into three groups according to their conditions: control group (*n* = 14,457), osteoporosis group (*n* = 913), sarcopenia group (*n* = 709), and osteosarcopenia group (*n* = 175). Table 1 shows descriptive characteristics of participants at the baseline, about demographics, health behaviors, chronic conditions, divided into four groups according to the presence or absence of osteosarcopenia. Participants affected by osteosarcopenia were significantly older and more frequently female than their counterparts not affected by this condition. Moreover, they were less frequently married and educated than people without sarcopenia. People with osteosarcopenia were more sedentary and suffered more frequently from all the chronic conditions reported in Table 1, except for Parkinson's disease. Finally, participants with osteosarcopenia reported the highest levels in EURO-D scale, among all the groups, even if not affected by depression at the baseline. The parameters to define osteosarcopenia (SMM, SMI, and handgrip strength) weakly correlate with the values of EURO-D at the baseline (all analyses: *p* < 0.0001; ASM: R = -0.13; SMI: R = -0.15; handgrip strength: R = -0.17).

**Table 1:** Descriptive baseline characteristics by the presence or absence of osteoporosis, sarcopenia and osteosarcopenia.

Parameter	Controls (***n*** = 14,457)	Only osteoporosis (***n*** = 913)	Only sarcopenia (n = 709)	Osteosarcopenia (n = 175)	p-Value
Demographics					
Mean age (SD)	62.9 (9.3)	67.0 (9.1)	72.8 (10.1)	74.9 (8.6)	<0.0001
Females (%)	6649 (46)	709 (77.7)	698 (98.4)	174 (99.4)	<0.001
Married and living together with spouse (%)	11,189 (77.4)	633 (69.3)	388 (54.7)	84 (48.0)	<0.0001
No educational title (%)	478 (3.3)	37 (4.1)	73 (10.3)	19 (10.9)	<0.0001

Health behaviors					
Sports or activities that are vigorous, hardly or never (%)	803 (5.6)	83 (9.1)	106 (15)	35 (20)	<0.0001
Drinking >2 glasses of alcohol every of 5/6 days/week (%)	424 (2.9)	21 (2.3)	10 (1.4)	3 (1.7)	<0.0001
High physical activity level (%)	6049 (41.8)	297 (32.5)	136 (19.2)	32 (18.3)	<0.0001
Current smoking (%)(%)	2927 (20.2)	157 (17.2)	53 (7.5)	8 (4.6)	<0.0001
EURO-D	1.19 (1.06)	1.41 (1.06)	1.60 (1.08)	1.77 (1.00)	<0.0001

Chronic conditions					
Heart attack (%)	1370 (9.5)	102 (11.2)	86 (12.1)	32 (18.3)	<0.0001
High blood pressure (%)	4084 (28.2)	312 (34.2)	290 (40.9)	73 (41.7)	<0.0001
High blood cholesterol (%)	2800 (19.4)	257 (28.1)	158 (22.3)	45 (25.7)	<0.0001
Stroke (%)	307 (2.1)	36 (3.9)	28 (3.9)	9 (5.1)	<0.0001
Diabetes (%)	1149 (7.9)	73 (8)	101 (14.2)	17 (9.7)	<0.001
COPD (%)	475 (3.3)	61 (6.7)	36 (5.1)	5 (2.9)	<0.0001
Asthma (%)	482 (3.3)	42 (4.6)	37 (5.2)	10 (5.7)	0.004
Arthritis (%)	1814 (12.5)	224 (24.5)	229 (32.3)	68 (38.9)	<0.0001
Cancer (%)	594 (4.1)	62 (6.8)	37 (5.2)	17 (9.7)	<0.0001
Stomach or duodenal ulcer (%)	665 (4.6)	64 (7)	25 (3.5)	15 (8.6)	<0.0001
Parkinson's disease (%)	33 (0.2)	4 (0.4)	2 (0.3)	2 (1.1)	0.68
Cataract (%)	707 (4.9)	91 (10)	96 (13.5)	37 (21.1)	<0.0001

Note: Data are reported as means with standard deviation (SD) for continuous variables and numbers and corresponding percentages for categorical factors.

**Fig. 2:**
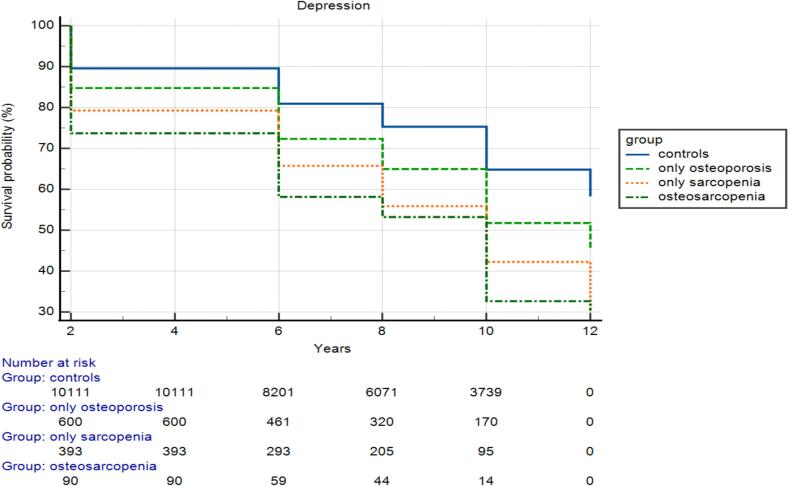
Risk of incident depression, according to the presence of osteosarcopenia at the baseline.

During the follow-up period of 12 years, 5056 participants (31.1 % of the initial population) became depressed. As shown in Fig. 2, during the 12 years of follow-up, people affected by osteosarcopenia became depressed in more than half of the cases compared to a quarter of controls. Moreover, in mean a participant affected by osteosarcopenia became depressed in 7.8 years, a control in 10 years. Table 2 shows the association between the presence or absence of osteosarcopenia at the baseline and the incidence of depression. In the basic model (i.e., adjusted for age and sex), all the groups were associated with a higher risk of depression. However, after adjusting for several potential baseline confounding variables, only sarcopenia (HR = 1.17; 95 % CI 1.04–1.32; *p* = 0.009) and osteosarcopenia (HR = 1.27; CI 95 % 1.12–1.58; *p* = 0.003) were significantly associated with a higher risk of depression. In a linear regression analysis, using as dependent variable the values of EURO-D score at the longest evaluation possible and as covariates those indicated in the multivariate Cox's regression analysis, including the changes of EURO-D scores during the follow-up period, we found that higher baseline handgrip values were associated with lower EURO-D scores during the follow-up period (beta = −0.07; 95%CI: −0.02 to −0.005; *p* = 0.001), where SMM (*p* = 0.61) and SMI (*p* = 0.24) were not.)

**Table 2:** Association between osteosarcopenia at the baseline and incident depression.

Labels	Number of events	Number of participants	Cumulative incidence	Basic model	p-value	Fully-adjusted model	p-Value
Controls	4275	14,457	29.6	1		1	
Only osteoporosis	360	913	39.4	1.14 (1.02–1.28)	0.02	1.08 (0.96–1.20)	0.20
Only sarcopenia	333	709	47.0	1.25 (1.11–1.40)	<0.0001	1.17 (1.04–1.32)	0.009
Osteosarcopenia	88	175	50.3	1.40 (1.13–1.74)	0.002	1.27 (1.12–1.58)	0.003

Note: Data are reported as hazard ratios and their corresponding 95 % confidence intervals. Analyses were adjusted for baseline values of country (dummy variable), age, sex, marital status, educational level, current smoking, drinking habits, physical activity level and presence of baseline heart attack, high blood pressure, high blood cholesterol, stroke, diabetes, COPD, asthma, arthritis, cancer, stomach or duodenal ulcer, Parkinson's disease, cataract.

Finally, to test the robustness of our results, we performed several sensitivity analyses using the factors cited before as strata (for age, we used the median value). Interaction among all of these factors were not statistically significant. Each increase in one SD of SMM was associated with a significant reduction in depression of 3 % (HR = 0.97, 95%CI: 0.95–0.97; *p* < 0.0001).

## 4 Discussion

To the best of our knowledge, this is one of the first studies to analyze the association between osteosarcopenia and depression in older people in a longitudinal fashion. Our findings support the hypothesis that osteosarcopenia increases the risk of depression, as participants with osteosarcopenia at baseline were significantly more likely to develop depression over the 12-year follow-up. This temporal association suggests that musculoskeletal deterioration may contribute to the onset of depressive symptoms, rather than simply being a consequence of pre-existing depression. In the present study, 175 participants (1.05 %) were categorized as having osteosarcopenia. This finding is somewhat in line with the previous literature. For example, a study carried out in Australia on a sample of 2353 participants found that osteosarcopenia increased with age for men from 14.3 % (60–64 years) to 59.4 % (≥75 years) and for women from 20.3 % (60–64 years) to 48.3 % (≥75 years) (Kirk et al., 2020). However, the study included a relatively small community-dwelling population, mean age of the population included in the current study was 67 years.

The public health burden of depression in older adults is reflected in suffering and disability, increased caregiving weight for family members, greater utilization of healthcare resources as a result of depression not being recognized or adequately treated, increased risk of all-cause mortality and suicide, and higher risk of Alzheimer's disease and vascular dementia (Reynolds III et al., 2019). In the present study we found that those with sarcopenia and osteosarcopenia had a higher risk of depression of 17 % and 27 %, respectively, compared to participants not affected by these conditions, even after adjusting for several potential confounders at the baseline. It should be acknowledged that the 95 % CI of sarcopenia alone and osteosarcopenia overlaps, suggesting that osteosarcopenia is associated with the onset of depression, owing to the presence of sarcopenia. Moreover, it is relevant to indicate that people affected by osteosarcopenia became depressed in over half of the cases compared to a quarter of controls and that in mean a participant affected by osteosarcopenia became depressed 2 years before the controls. These data clearly indicate an important association between osteosarcopenia and depression in older people.

Findings from the present study support those of previous literature. For example, a study carried out in China on a sample of 4652 participants demonstrated that sarcopenia was an independent predictor for the occurrence of depressive symptoms, however, osteosarcopenia was not studied (Gao et al., 2021). Another study carried out in Asia on a sample of 337 participants found that those with osteosarcopenia had higher levels of depressive symptoms compared to those who did not. However, there was no significant difference in depressive mood and the study reported that the method used to measure sarcopenia was not reliable (Chou et al., 2023).

A possible explanation for the association between osteosarcopenia and depression is that osteosarcopenia is often linked to the symptoms of depression, which could be caused by the adverse consequences of physical health on mental health (Chang et al., 2017). Additionally, depression might also be connected to a decline in physical function, thus aggravating the symptoms of osteosarcopenia and vice versa (Polito et al., 2022). Deterioration of physical function attributable to bone and muscle deprivation may induce social frailty and decrease activity, contributing to depression. Moreover, depression is associated with lower physical activity levels, distressed hormone levels, and altered bone metabolism, possibly exacerbating osteoporosis and sarcopenia. This double link makes a composite sequence of interactions (Polito et al., 2022; Papadopoulou et al., 2021). A study carried out in Canada on 8888 participants found that osteosarcopenia was associated with multiple negative outcomes, such as self-reported incident falls and fractures in males and worse life satisfaction and ADL for all participants (Lee et al., 2024). Indeed, findings from the present study further indicate that osteosarcopenia increases the risk of depression in older adults, underscoring the necessity to screen those with this condition for depression in daily clinical practice. From a practical point of view, it was reported that a combined multidisciplinary approach combining physical exercise, particularly resistance and protein supplementation could be promising to reverse both osteosarcopenia and depression (Yoshimura et al., 2025; Whaikid and Piaseu, 2024; Gordon et al., 2018; Rossi et al., 2024). Therefore, our study could further indicate the need to assess depression in people suffering from osteosarcopenia and early propose tailored interventions.

### 4.1 Limitations

The present study should be interpreted within the confines of several limitations. Firstly, objective measures of body composition were not performed. Therefore, we used surrogates of low fat-free mass based on anthropometric parameters. Though a direct assessment of body composition was not done, the equation suggested by Lee and colleagues has a good agreement with the gold standard tool for evaluating body composition, i.e. DXA (González-Mendoza et al., 2019). Secondly, even if EURO-D is considered a validated score for older people (Prince et al., 1999b), it describes only depressive symptoms, without a diagnosis of major depressive disorder according to DSM V (e.g., did not consider medications or other characteristics of depression). Osteoporosis and previous fractures were self-reported and not confirmed by X-rays or bone densities, respectively. Finally, some variables such as those concerning medical conditions were self-reported, which could introduce a level of both social and desirability bias.

## 5 Conclusions

The present study found a significant association between osteosarcopenia and incident depression, over 12 years of follow-up, mainly driven by the presence of sarcopenia. If future research confirms the present findings, it may then be prudent to target those with osteosarcopenia to aid in the prevention of onset depression.

## CRediT authorship contribution statement

**Nicola Veronese:** Writing – original draft. **Francesco Saverio Ragusa:** Data curation. **Shaun Sabico:** Writing – original draft. **Ligia Juliana Dominguez:** Writing – review & editing. **Mario Barbagallo:** Writing – review & editing. **Gustavo Duque:** Writing – review & editing. **Lee Smith:** Formal analysis, Writing – original draft. **Nasser Al-Daghri:** Writing – review & editing.

## Sponsor's role

The sponsor supported the data analysis of paper.

## Funding sources

This work was supported by the Deanship of Scientific Research, KSU and Chair for Biomarkers of Chronic Diseases (CBCD). The SHARE data collection has been funded by the 10.13039/501100000780European Commission through FP5 (QLK6-CT-2001-00360), FP6 (SHARE-I3: RII-CT-2006-062193, COMPARE: CIT5-CT-2005-028857, SHARELIFE: CIT4-CT-2006-028812), FP7 (SHARE-PREP: GA N°211909, SHARE-LEAP: GA N°227822, SHARE M4: GA N°261982, DASISH: GA N°283646) and 10.13039/100010661Horizon 2020 (SHARE-DEV3: GA N°676536, SHARE-COHESION: GA N°870628, SERISS: GA N°654221, SSHOC: GA N°823782) and by DG 10.13039/501100000893Employment, Social Affairs, and Inclusion. Additional funding from the German Ministry of Education and Research, the Max Planck Society for the Advancement of Science, the 10.13039/100000049U.S. National Institute on Aging (U01_AG09740-13S2, P01_AG005842, P01_AG08291, P30_AG12815, R21_AG025169, Y1-AG-4553-01, IAG_BSR06-11, OGHA_04-064, and HHSN271201300071C) and from various national funding sources is gratefully acknowledged (see www.share-project.org).

## Declaration of competing interest

None.

## Data Availability

The data are available upon request to the corresponding author. The data were not presented in any national or international conference.
